# Efficacy of CDK4/6 inhibitors combined with endocrine therapy in HR+/HER2− breast cancer: an umbrella review

**DOI:** 10.1007/s00432-023-05516-1

**Published:** 2024-01-19

**Authors:** Dongqing Pu, Debo Xu, Yue Wu, Hanhan Chen, Guangxi Shi, Dandan Feng, Mengdi Zhang, Zhiyong Liu, Jingwei Li

**Affiliations:** 1 First Clinical Medical College, Shandong University of Traditional Chinese Medicine, Jinan, China; 2 College of Traditional Chinese Medicine, Shandong University of Traditional Chinese Medicine, Jinan, China; 3 Breast Thyroid Surgery, Affiliated Hospital of Shandong University of Traditional Chinese Medicine, Jinan, 250014 Shandong China; 4 Institute for Literature and Culture of Chinese Medicine, Shandong University of Traditional Chinese Medicine, Jinan, China; 5 Central Laboratory, The Affiliated Hospital of Shandong University of Traditional Chinese Medicine, Jinan, China

**Keywords:** CDK4/6 inhibitor, Endocrine therapy, Breast cancer, Clinical efficacy, Umbrella review, Meta-analysis and systematic review

## Abstract

**Background:**

The use of Cyclin-Dependent kinase 4 and 6 (CDK4/6) inhibitors has profoundly changed the challenge of endocrine therapy (ET) resistance in hormone receptor-positive (HR+)/HER2-negative (HER2−) breast cancer. However, there is currently no comprehensive evaluation of the evidence for the efficacy of CDK4/6 inhibitors. We conducted an umbrella review to explore the impact of CDK4/6 inhibitor combined with ET on breast cancer by summarizing and assessing the meta-analysis (MA) and systematic review (SR) evidence.

**Methods:**

Cochrane, PubMed, Embase, and Web of Science databases were searched from inception to August 1st, 2022. Eligible studies were assessed for methodological quality, report quality, and evidence quality using the AMSTAR-2 scale, PRISMA 2020, and GRADE grading systems, respectively. We summarized all efficacy outcomes of CDK4/6 inhibitors for breast cancer and reported them in narrative form.

**Results:**

Our study included 24 MAs and SRs. The strongest evidence demonstrated that CDK4/6 inhibitor combined with ET significantly improved progression-free survival (PFS), overall survival (OS) in advanced breast cancer (ABC). A large body of moderate to high evidence showed a significant association between combination therapy and objective response rate (ORR), and clinical benefit response (CBR) benefit in ABC. Low evidence suggested some degree of benefit from combination therapy in second progression-free survival (PFS2) and time to subsequent chemotherapy (TTC) outcomes in ABC and invasive disease-free survival (IDFS) outcomes in early breast cancer.

**Conclusions:**

Based on current evidence, CDK4/6 inhibitors combined with ET have great confidence in improving PFS, OS, ORR, and CBR outcomes in patients with ABC, which provides more rational and valid evidence-based medicine for CDK4/6 inhibitor promotion and clinical decision support.

**Supplementary Information:**

The online version contains supplementary material available at 10.1007/s00432-023-05516-1.

## Introduction

Due to its high morbidity and death rates, breast cancer is one of the most difficult malignancies for women to treat worldwide. Globally, there were 2.3 million new instances of breast cancer (11.7%) and 585,000 deaths from the disease (6.9%), according to GLOBOCAN 2020, a compilation of cancer statistics from 185 countries (Sung et al. 2021). The most prevalent molecular subtype of breast cancer is hormone receptor-positive (HR+)/HER2-negative (HER2−) breast cancer (Lin et al. 2022). Endocrine therapy (ET) is the preferred treatment for patients with HR+ advanced breast cancer (ABC) who do not have visceral crises or other serious diseases, and it is the primary adjuvant therapy for patients with HR+ early breast cancer (EBC) 5–10 years after surgery (Hong and Xu 2022; Andre et al. 2022). Nevertheless, despite the obvious clinical benefit of ET, about 25% of patients with EBC and almost all patients with metastatic breast cancer (MBC) develop primary or secondary drug resistance, which in turn causes disease progression and recurrent metastasis, posing a significant challenge to clinicians (Jeselsohn et al. 2015).

Cyclin-dependent kinases (CDKs) were discovered to be members of a wide family of serine/threonine protein kinases that control cell cycle progression as a result of developments in molecular biology and our growing understanding of breast cancer. In particular, cyclin D bind to CDK4 and CDK6, induce hyperphosphorylation of retinoblastoma protein (Rb), promote E2F-mediated cell cycle gene transcription, and promotes tumor cell progression from the G1 to S phase of the cell cycle, which promotes breast cancer cell proliferation a pathway closely associated with ET resistance in patients with HR+ breast cancer (Roberto et al. 2021; Lloyd et al. 2022; Alves et al. 2021). By specifically inhibiting the cyclin D-CDK4/6-Rb pathway, CDK4/6 inhibitors overcome endocrine resistance in HR+ breast cancer, which successfully delays the progression of the disease (Roberto et al. 2021; Huang et al. 2022). The considerable therapeutic effect of CDK4/6 inhibitors in conjunction with ET has been demonstrated in several randomized controlled trials (RCTs) for HR+/HER2− EBC and ABC (Johnston et al. 2020; Mayer et al. 2021; Loibl et al. 2021; Finn et al. 2015, 2016). In light of promising data from clinical trials, The Food and Drug Administration (FDA) approved three CDK4/6 inhibitors, palbociclib, ribociclib, abemaciclib, for use in the first and second-line treatment of HR+/HER2− MBC (Mullard 2017; Burstein et al. 2021). Besides, abemaciclib was given FDA approval in October 2022 for use in conjunction with ET adjuvant treatment in persons with EBC who had HR+/HER2-, Ki-67 ≥ 20%, lymph node positivity, and high risk of recurrence (Royce et al. 2022).

Clinical efficacy assessment is the key to clinical decision-making for CDK4/6 inhibitors. With the gradual disclosure of RCTs for CDK4/6 inhibitors in combination with ET in patients with HR+/HER2− breast cancer, an increasing number of MAs and SRs are summarizing and analyzing clinical efficacy from multiple perspectives. But these MAs and SRs are different in evidence intensity, and not all data results can provide reliable evidence, which adds limited value to guide clinical practice. However, an umbrella review can make a broad overview, summary, and comparison of the research topics, and then evaluate and improve the evidence quality of evidence-based medicine (Bonczar et al. 2022; Wu et al. 2022). Therefore, we made an umbrella review. To our knowledge, this is the first comprehensive and critical summary of the top evidence for CDK4/6 inhibitors in combination with ET for breast cancer as a way to provide more rational and effective evidence-based medical evidence for clinical decision-making.

## Methods

### Protocol and registration

An umbrella review of the clinical efficacy of CDK4/6 inhibitors for the treatment of breast cancer patients was performed according to the Preferred Reporting Items for Systematic Evaluation and Meta-Analysis (PRISMA) program. The protocol has been previously published and registered in The International Prospective Register of Systematic Reviews (PROSPERO) database (CRD42022350167).

### Search strategy

From inception to August 1, 2022, the Cochrane, PubMed, Embase, and Web of Science databases were searched for relevant SRs and MAs. The key search terms were “breast cancer,” “Cyclin-Dependent Kinases 4 and 6 Inhibitors,” “systematic review,” and “meta-analysis,” and subject terms and free words for each database were combined using Boolean operators. English was selected as the language. The database search strategy is shown in the supplementary materials (Supplemental Table 2).

### Eligibility criteria

The selection criteria were based on participants, interventions, comparisons, outcomes, and study design (PICOS). Studies meeting all of the following criteria were considered eligible: (a) the population was breast cancer, regardless of race or age; (b) CDK4/6 inhibitor therapy combined with ET in the trial group and ET alone or combined with placebo in the control group; (c) providing key data (such as relative risk, advantage ratio, relative ratio, and risk ratio) and clinical efficacy outcomes. (d) The type of study was an SR and MA that included only RCTs.

Conversely, studies meeting at least one of the following criteria were excluded: (a) duplicate publications; (b) articles with incomplete reporting of key data; (c) umbrella review protocols or quality evaluations, conference abstracts, etc.

### Literature screening and data extraction

Two reviewers (WY and XDB) independently screened and extracted all records for literature selection, first removing duplicate literature, then screening titles and abstracts, and reading the full literature for further evaluation. Two reviewers independently extracted the following data from all eligible reviews using a standardized spreadsheet (Excel): first author, year of publication, country, study population, sample size, intervention/control measures, outcome indicators, quality assessment methods, effect intervals, *p* values, heterogeneity *I*^2^, etc. In the event of a disagreement, a third researcher (CHH) was consulted.

### Data analysis

Two independent evaluators (WY and XDB) evaluated the methodological quality, report quality, and evidence quality of eligible research using the Assessment of Multiple Systematic Reviews-2 (AMSTAR-2) scale, PRISMA statement, and the Grades of Recommendation, Assessment, Development, and Evaluation (GRADE) analysis, and resolved disagreements through third-party reviewer discussions (CHH). The methodological quality of the study was assessed using the AMSTAR-2 scale. The AMSTAR-2 consists of 16 items that the researcher evaluates as “yes”, “no”, and “partial yes” according to the degree of satisfaction with the evaluation criteria (Shea et al. 2017, 2009). The quality of reporting of the included studies was assessed using the PRISMA 2020 checklist (Hutton et al. 2015). The PRISMA 2020 statement consists of 27 items (42 level sub-entries), and the scoring principle is that each item fully reporting is scored as 1, partial reporting as 0.5, and non-reporting as 0, out of 42 points. < 25 is classified as having relatively serious information deficiencies, 25–32 as reporting some deficiencies, and 33–42 as reporting relatively complete (Page et al. 2021a, b). The GRADE system makes judgments about the quality of evidence-based on effect sizes and considers risk of bias, inconsistency, indirectness, precision, and publication bias. It grades the evidence as high, moderate, low, or very low (Zeng et al. 2021; Schunemann et al. 2020) (Supplemental Table 1).

**Table 1:** Main characteristics of systematic reviews and meta-analyses included in the present umbrella review

Serial number	Author(s), year	Country	Trials (subjects)	Population	Experimental intervention	Control intervention	Outcome investigated	Quality assessment	AMSTAR-2	PRISMA
1	Agostinetto et al. (2021)	Belgium	3 (12,647)	HR+/HER2− early breast cancer	CDK4/6is + ET	ET	IDFS, DRFS	Cochrane criteria	L	34
2	Ding et al. (2018)	China	6 (3182)	HR+/HER2− advanced breast cancer	CDK4/6is + ET	ET	PFS, ORR, CBR	Cochrane criteria	VL	28
3	Gao et al. (2021)	China	3 (12,647)	HR+/HER2− early breast cancer	CDK4/6is + ET	ET	IDFS	Cochrane criteria	VL	36
4	Guo et al. (2019)	China	3 (1352)	HR+/HER2− advanced breast cancer	palbociclib + ET	ET	PFS, OS	QUADAS‐2	VL	27
5	Lee et al. (2019)	Australia	4 (2499)	HR+/HER2− advanced breast cancer	CDK4/6is + ET	ET	PFS	–	VL	24.5
6	Li et al. (2020a, b)	China	8 (4580)	HR+/HER2− advanced breast cancer	CDK4/6is + ET	ET	PFS, OS, ORR, CBR	Cochrane criteria	VL	32
7	Li et al. (2020a, b)	China	9 (5043)	HR+/HER2− metastatic breast cancer	CDK4/6is + ET	ET	OS, PFS, ORR	–	VL	26
8	Li et al. (2021)	China	7 (4415)	HR+/HER2− advanced breast cancer	CDK4/6is + ET	ET	PFS, OS, ORR, CBR	Cochrane criteria	VL	25
9	Lin et al. (2020)	China	6 (3421)	HR+/HER2− metastatic breast cancer	CDK4/6is + ET	ET	OS	Cochrane criteria	VL	30
10	Messina et al. (2018)	Italy	8 (4578)	HR+/HER2− advanced breast cancer	CDK4/6is + ET	ET	PFS, ORR	Cochrane criteria	VL	26
11	Omarini et al. (2020)	Italy	4 (855)	HR+/HER2− advanced breast cancer	CDK4/6is + ET	ET	PFS	–	VL	26
12	Ramos-Esquivel et al. (2020)	Costa Rica	3 (1916)	HR+/HER2− metastatic breast cancer	CDK4/6is + fulvestrant	Fulvestrant	OS, PFS, ORR	Cochrane criteria	VL	28
13	Ramos-Esquivel et al. (2018)	Costa Rica	3 (1827)	post-menopausal HR+/HER2− metastatic breast cancer	CDK4/6is + AI	AI	PFS, ORR, CBR	Cochrane criteria	VL	29
14	Schettini et al. (2020)	Italy	6 (3421)	HR+/HER2− metastatic breast cancer	CDK4/6is + ET	ET	OS	Cochrane criteria	VL	28
15	Shimoi et al. (2020)	Japan	4 (1992)	post-menopausal HR+/HER2− metastatic breast cancer	CDK4/6is + AI	AI	PFS, ORR, CBR	Cochrane criteria	VL	27
16	Tian et al. (2021)	China	8 (4580)	HR+/HER2− advanced breast cancer	CDK4/6is + ET	ET	PFS, OS, ORR	Cochrane criteria	VL	30.5
17	Xu et al. (2020)	China	8 (4580)	HR+/HER2− advanced breast cancer	CDK4/6is + ET	ET	PFS, OS, ORR, CBR	Cochrane criteria	VL	29
18	Yang et al. (2021)	China	6 (3685)	HR+/HER2− advanced breast cancer	CDK4/6is + ET	ET	PFS	Cochrane criteria	L	30.5
19	Zheng et al. (2020)	China	9 (5043)	HR+/HER2− advanced breast cancer	CDK4/6is + ET	ET	PFS, OS, ORR, CBR	Cochrane criteria	VL	31.5
20	Munzone et al. (2021)	Italy	8 (4580)	HR+/HER2− metastatic breast cancer	CDK4/6is + ET	ET	PFS2, TTC, PFS, OS	–	VL	23.5
21	Piezzo et al. (2020)	Italy	8 (4580)	HR+/HER2− metastatic breast cancer	CDK4/6is + ET	ET	PFS, OS, ORR	Cochrane criteria	L	36
22	Toss et al. (2019)	Italy	4 (2499)	HR+ Bone-Only Metastatic Breast Cancer	CDK4/6is + ET	ET	PFS	–	VL	26
23	Wang et al. (2020)	China	5 (2695)	HR+/HER2− advanced breast cancer	CDK4/6is + ET	ET	OS, PFS, ORR	–	VL	15.5
24	Deng et al. (2018)	China	7 (3854)	HR+/HER2− advanced breast cancer	CDK4/6is + ET	ET	PFS, ORR	Cochrane criteria	VL	26.5

*CDK4/6is* CDK4/6 inhibitors, *ET* endocrine therapy, *AI* aromatase inhibitors, *IDFS* invasive disease-free survival, *DRFS* distant relapse-free survival, *PFS* progression-free survival, *OS* overall survival, *ORR* objective response rate, *CBR* clinical benefit response, *PFS2* second progression-free survival, *TTC* time to subsequent chemotherapy, *AMSTAR-2* Assessment of Multiple Systematic Reviews-2, *PRISMA* preferred reporting items for systematic reviews and meta-analyses, *L* low, *VL* very low

As recommended by the Joanna Briggs Institute for Umbrella Reviews, a descriptive analysis of outcome indicators for CDK4/6 inhibitors combined with ET for breast cancer was carried out; no data reanalysis was carried out. We pooled summary indicators (risk ratio (HR), relative risk (RR), odds ratio (OR), risk difference (RD), and 95% confidence interval (CI)) for CDK4/6 inhibitors. The *I*^2^ statistic was used to describe the heterogeneity between studies. Statistical significance was defined as *P* values < 0.05. The breast cancer population was divided into EBC and ABC in order to provide additional details on CDK4/6 inhibitors in breast cancer patients.

## Results

### Literature search

**Fig. 1:**
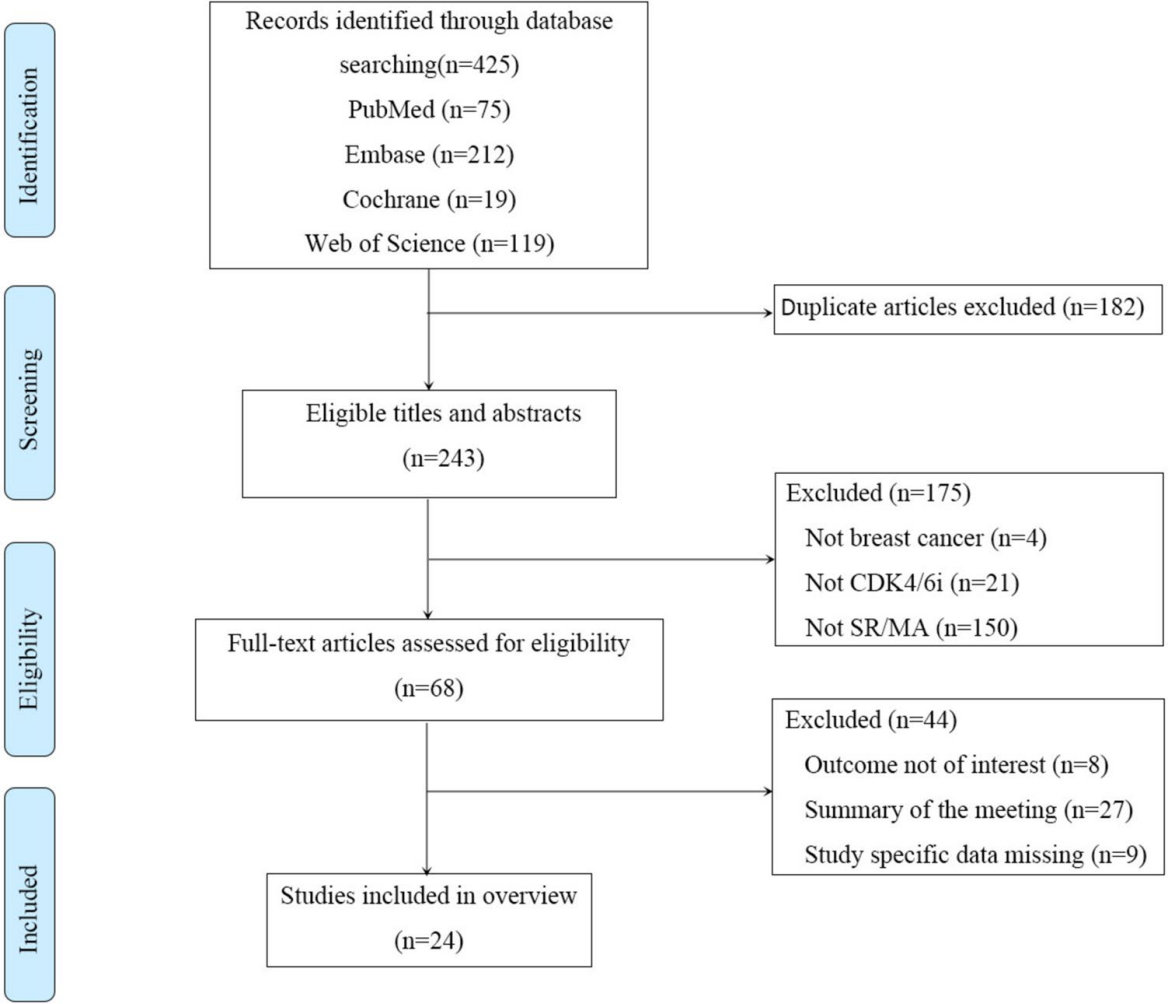
The flowchart of the literature screening. *SR* systematic review, *MA* meta-analysis

By searching 4 databases, a total of 425 pertinent papers were found; 243 remained after duplicates were eliminated. Following title, abstract, and full-text screening, 219 pertinent papers were eliminated, of which 27 were conference abstracts. The inclusion criteria were met by a total of 24 papers. The findings of the literature screening process are displayed (Fig. 1).

### Basic characteristics of the included literature

All included literature was published during 2018–2022, and the number of included literature included in SR/MA ranged from 3 to 9 with sample sizes between 855 and 12,647 (Agostinetto et al. 2021; Gao et al. 2021; Li et al. 2020a, b, 2021; Tian et al. 2021; Yang et al. 2021; Munzone et al. 2021; Lin et al. 2020; Ramos-Esquivel et al. 2020, 2018; Xu et al. 2020; Zheng et al. 2020; Shimoi et al. 2020; Piezzo et al. 2020; Wang et al. 2020; Omarini et al. 2020; Schettini et al. 2020; Lee et al. 2019; Toss et al. 2019; Guo et al. 2019; Ding et al. 2018; Messina et al. 2018; Deng et al. 2018). Two of these trials (serial number: 1, 3) included populations of HR+/HER2− EBC and the remainder were HR+/HER2− ABC (Agostinetto et al. 2021; Gao et al. 2021). In terms of therapeutic drugs, mostly CDK4/6 inhibitors combined with ET versus ET, one study (serial number: 12) (Ramos-Esquivel et al. 2020) reported CDK4/6 inhibitors combined with fulvestrant versus fulvestrant alone as an intervention versus control, and 2 studies (serial number: 13, 15) reported CDK4/6 inhibitors combined with aromatase inhibitors (AI) versus AI alone (Shimoi et al. 2020; Ramos-Esquivel et al. 2018). For patients with EBC, invasive disease-free survival (IDFS) was the primary outcome indicator, whereas progression-free survival (PFS), overall survival (OS), objective response rate (ORR), and clinical benefit response (CBR) outcomes were mostly employed for patients with ABC. 17 papers (serial number: 2–4, 6, 8–10, 12–19, 21, 24) utilized the Cochrane criteria assessment technique for risk of bias assessment, 1 article used QUADAS-2 (serial number: 4) (Guo et al. 2019), and 6 studies (serial number: 5, 7, 11, 20, 22, 23) did not disclose their risk of the bias assessment method (Munzone et al. 2021; Li et al. 2020a, b; Wang et al. 2020; Omarini et al. 2020; Lee et al. 2019; Toss et al. 2019). Characteristics of the included studies are reported (Table 1).

### Methodological quality from included studies

**Fig. 2:**
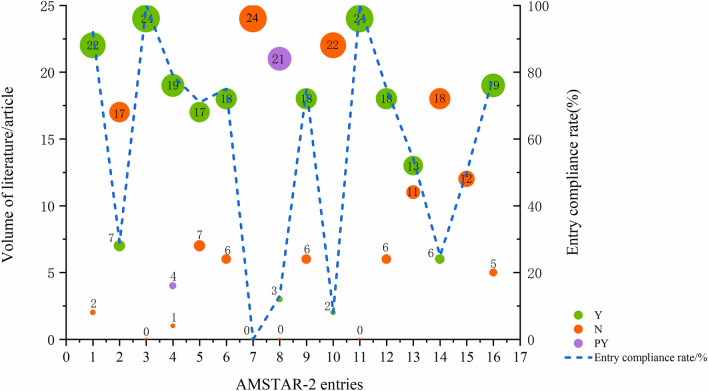
Results of the AMSTAR-2 assessment. *Y* Yes, *N* No, *PY* Partial Yes. Each entry is Y for full compliance, PY for partial compliance, and N for non-compliance; entry compliance rate = (number of documents complying with this entry/total documents) × 100%

The methodological quality of the included studies was assessed by the AMSTAR-2 scale. Three articles (serial number: 1, 18, 21)’ quality was classified as low quality, and the remainder (serial number: 2–17, 19, 20, 22–24) as very low quality. Of the 16 entries, Entries 3 and 11 had a 100% attainment rate, all specifying the type of literature included in the study and using appropriate statistical methods for the combined analysis of results. Entries 7, 10, and 8 had lower attainment rates of 0%, 8%, and 13%, respectively. This indicated that the MA and SR had significant issues with providing a list of excluded literature with justifications for exclusion, reporting the funding sources of included studies and providing a detailed description of the fundamental characteristics (Table 1, Fig. 2, and Supplemental Table 3).

### Quality of reports from included studies

**Fig. 3:**
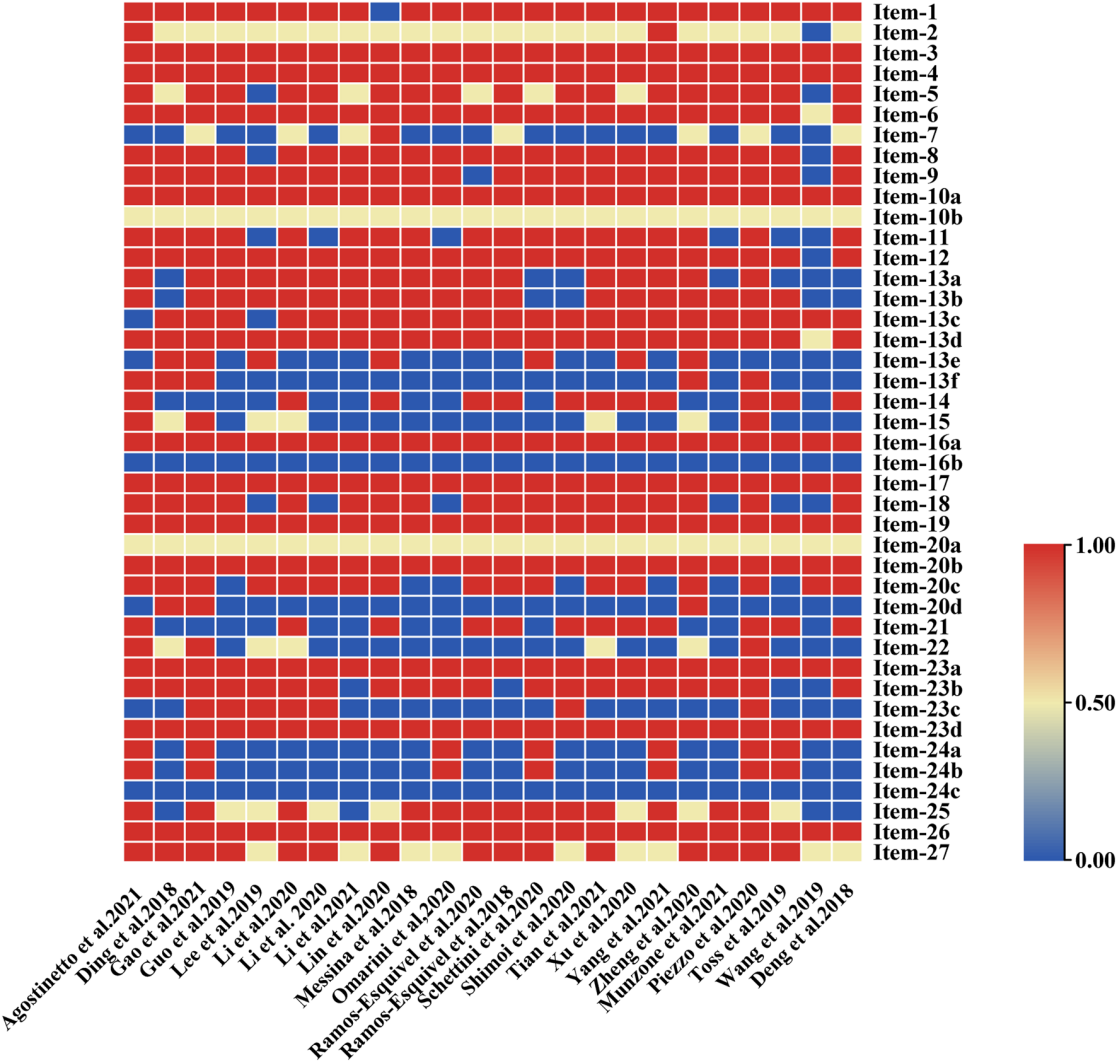
Results of the PRISMA assessment. Each of the PRISMA entries vertically and 24 documents horizontally scored one point for complete reporting (colored red), 0.5 points for partial reporting (colored yellow), and 0 points for no reporting (colored blue)

The quality of the 24 publications included was evaluated using the PRISMA 2020 report, with scores ranging from 15.5 to 35 and a mean score of 27.1. Among them, there were 3 reports (serial number: 1, 3, 21) with scores of 33 to 42, and the reports were relatively complete. 18 reports (serial number: 2, 4, 6–19, 22, 24) with scores of 25 to 32 had some defects. There were 3 reports (serial number: 5, 20, 23) with scores of < 25, and the reports had serious defects. Of the 42 sub-entries reported in PRISMA, the main ones reporting significant missing information were 7, 13e, 13f, 16b, 20d, 23c, 24a, 24b, and 24c, which involved incomplete search strategies, lack of methods to analyze the heterogeneity and sensitivity analyses, failure to explain the rationale for excluding data from study selection, no sensitivity analysis was performed in the synthesis results, the discussion that did not describe any limitations of the review process, and failure to provide registration and protocol-related information (Table 1, Fig. 3 and Supplemental Table 4).

### Evaluation of the quality of evidence from included studies

A total of 3 EBC and 55 ABC efficacy indicators were obtained for this study, and assessment using GRADE revealed evidence quality for each indicator. Most of the evidence quality was concentrated in moderate (20 items, approximately 34.48%) and low (24 items, approximately 41.38%), while the remaining evidence quality was high (6 items, approximately 10.34%) and very low (8 items, approximately 13.79%), respectively. Overall, the evidence quality was reduced mostly due to the risk of bias and publication bias of the RCTs in the original studies, which made the authenticity of the study results affected. Notably, for the ORR and CBR outcomes, inconsistency due to heterogeneity was also an important reason for downgrading (Supplemental Table 5).

### The efficacy of CDK4/6 inhibitors in the treatment of breast cancer

#### Early breast cancer

**Fig. 4:**
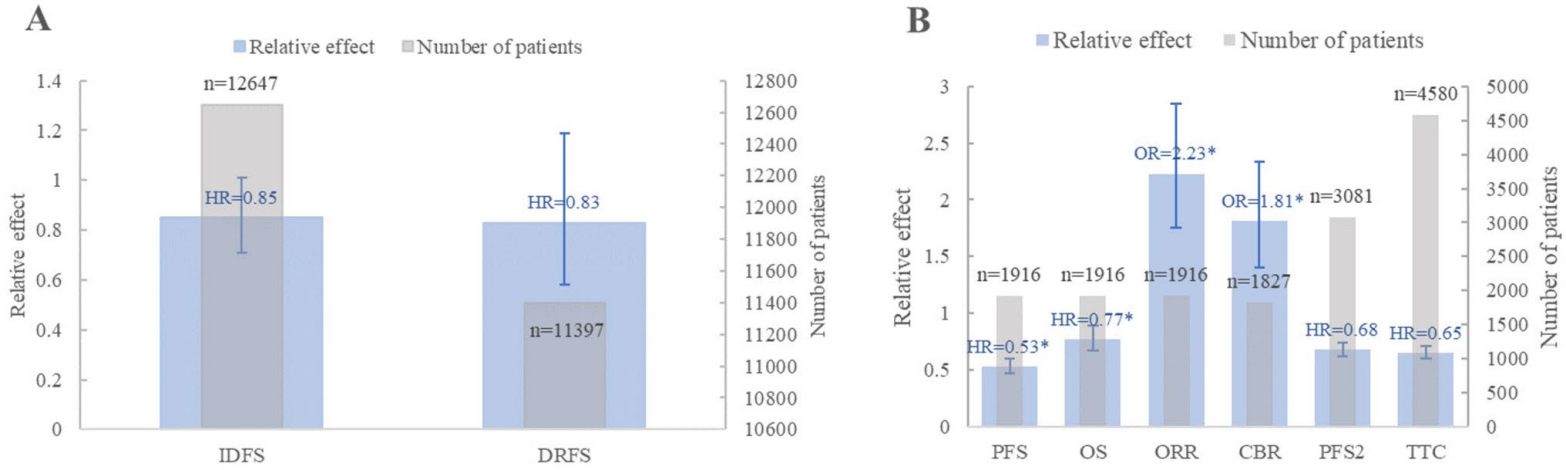
Summary of evidence for the association of CDK4/6 inhibitor with early breast cancer outcomes in systematic reviews with meta­analyses categorized as the most comprehensive for each outcome. **A** Summary of the efficacy of early breast cancer; **B** summary of the efficacy of advanced breast cancer

In 2 EBC meta-analyses (serial number: 1, 3), the IDFS and Distant relapse-free survival (DRFS) outcome of CDK4/6 inhibitors coupled with ET against ET alone were compared (Agostinetto et al. 2021; Gao et al. 2021). Agostinetto E and Gao HF evaluated IDFS efficacy indicators in 12,647 patients with HR+/HER2− EBC and concluded that CDK4/6 inhibitors combined with ET had more IDFS benefit independent of tumor size, TNM stage, tumor stage, nodal stage, histologic grade, prior neoadjuvant chemotherapy, age, race, and menopausal status, but all had low quality of evidence. Remarkably, Agostinetto E and Gao HF disagreed on whether combination therapy was statistically significant in the benefit of IDFS; Gao HF highlighted a significant benefit of combination therapy and a statistically significant IDFS benefit was observed in the subgroup analysis of N2/N3 nodal stage (HR 0.83, 95% CI 0.71–0.97, *P* = 0.09). In contrast, the Agostinetto E results suggested a *P* value of > 0.05 for IDFS and consistent benefit in N0/N1 versus N2/N3 nodal stage (Agostinetto et al. 2021; Gao et al. 2021). For the DRFS outcome, CDK4/6 inhibitors combined with ET did not provide a meaningful effect and the quality of the evidence was low (Agostinetto et al. 2021) (Fig. 4A and Supplemental Table 5).

#### Advanced breast cancer

22 studies (serial number: 2, 4–24) investigated outcomes in ABC, comprising PFS, OS, ORR, CBR, second progression-free survival (PFS2), and time to subsequent chemotherapy (TTC). This study summarized and charted the outcomes with the highest quality of evidence and more comprehensive data on the efficacy outcomes (Fig. 4B and Supplemental Table 5).

##### PFS

A total of 20 papers (serial number: 2, 4–8, 10–13, 15–24) focused on PFS outcomes with a mixed quality of evidence (Li et al. 2020a, b, 2021; Tian et al. 2021; Yang et al. 2021; Munzone et al. 2021; Ramos-Esquivel et al. 2020, 2018; Xu et al. 2020; Zheng et al. 2020; Shimoi et al. 2020; Piezzo et al. 2020; Wang et al. 2020; Omarini et al. 2020; Lee et al. 2019; Toss et al. 2019; Guo et al. 2019; Ding et al. 2018; Messina et al. 2018; Deng et al. 2018), with 50% (10 items) of the evidence level certainty found to be middle to high evidence and the remaining evidence level low to very low. The middle to high evidence suggested that PFS was significantly better with CDK4/6 inhibitors in combination with fulvestrant, aromatase inhibitors, or other ET agents than with ET alone for both postmenopausal HR+/HER2− breast cancer and MBC patients, and all with zero heterogeneity, indicating the broad consistency and reliability of multiple investigations. Subgroup analysis showed that improved PFS was observed with both CDK4/6 inhibitors combined with ET compared to ET alone regardless of histopathological classification, endocrine resistance status, estrogen, and progesterone receptor status, site and number of tumor metastases, menopausal status, race, age, prior chemotherapy treatment, ET regimen, advanced disease treatment line, CDK4/6 inhibitor type, disease-free interval (DFI), treatment-free interval (TFI), etc. (Li et al. 2020a, b, 2021; Tian et al. 2021; Zheng et al. 2020; Piezzo et al. 2020; Lee et al. 2019; Ding et al. 2018; Messina et al. 2018; Ramos-Esquivel et al. 2018; Deng et al. 2018).

##### OS

OS results were reported by 13 MAs (serial number: 4, 6–9, 12, 14,16, 17, 19–21, 23) (Gao et al. 2021; Tian et al. 2021; Munzone et al. 2021; Li et al. 2020a, b; Lin et al. 2020; Ramos-Esquivel et al. 2020; Xu et al. 2020; Zheng et al. 2020; Piezzo et al. 2020; Wang et al. 2020; Schettini et al. 2020; Guo et al. 2019; Deng et al. 2018). The evidence quality ranged from middle to high for 38% (5 items). The findings implied that CDK4/6 inhibitor coupled with ET had a better OS than ET, all with zero heterogeneity. Eight studies were analyzed in subgroups based on stratified variables including PR status, menopausal status, the location and number of tumor metastases, age, race, line of disease treatment, endocrine therapeutic agents, and endocrine sensitivity status. The results demonstrated that CDK4/6 inhibitor combined with ET was superior to ET alone, with benefits consistent across subgroups (Tian et al. 2021; Li et al. 2020a, b; Lin et al. 2020; Ramos-Esquivel et al. 2020; Zheng et al. 2020). Particularly noteworthy were the CDK4/6 inhibitor-type subgroup analysis results. Regarding the stratification aspect of CDK4/6 inhibitor medications, Lin et al. (2020), Zheng et al. (2020), Piezzo et al. (2020), and Tian et al. (2021) all came to the conclusion that the OS of Ribociclib and abemaciclib in combination with ET was considerably better than ET therapy alone, however, no significant improvement was detected with palbociclib.

##### ORR

13 studies (serial number: 2, 6–8, 10, 12, 13, 15–17, 19, 21, 23, 24) reported ORR events in the CDK4/6 inhibitor plus ET group versus the ET alone group (Li et al. 2020a, b, 2021; Tian et al. 2021; Ramos-Esquivel et al. 2020, 2018; Xu et al. 2020; Zheng et al. 2020; Shimoi et al. 2020; Wang et al. 2020; Ding et al. 2018; Messina et al. 2018; Deng et al. 2018). It is notable that approximately 61% (8 items) of the evidence was of middle to high quality, implying greater confidence that the estimated results represent true treatment effects. Pooled data suggested that the addition of CDK4/6 inhibitor to ET-based therapy was associated with a statistically significant benefit in ORR independent of advanced disease treatment lines, menopausal status, CDK4/6 inhibitor type, or endocrine resistance status (Tian et al. 2021; Li et al. 2020a, b; Messina et al. 2018).

##### CBR

About 43% (3 items) of the data from 7 MAs (serial number: 2, 6, 8, 13, 15, 17, 19) that looked at CBR outcomes was of middle to high quality (Li et al. 2020a, b, 2021; Xu et al. 2020; Zheng et al. 2020; Shimoi et al. 2020; Ding et al. 2018; Ramos-Esquivel et al. 2018). The findings indicated a significant increase in CBR when CDK4/6 inhibitor and ET were coupled, but the heterogeneity was not yet uniform and the quality of research needed to be raised.

##### PFS2 and TTC

PFS2 stands for the time from the start of the randomization group to the second disease progression or death, and TTC is for the time to delayed chemotherapy. Munzone et al. (2021) analyzed the association of CDK4/6 inhibitor with PFS2 and TTC outcomes in metastatic breast cancer. The results suggested that CDK4/6 inhibitor plus ET improved PFS2 and TTC compared with ET alone, but statistically significant results for PFS2 and TTC were lacking in the literature and the quality of evidence was all low, so there was uncertainty about the clinical relevance of PFS2 to TTC outcomes with CDK4/6 inhibitor.

## Discussion

Our review identified 24 MAs and SRs and assessed the quality of evidence for 58 outcomes. Our data suggested that for patients with HR+/HER2− ABC, a large body of middle to high evidence was found to support that CDK4/6 inhibitor combined with ET significantly improved patients’ PFS, OS, ORR, and CBR compared with ET alone, demonstrating the reliability of the results. Subgroup analysis of ABC suggested that palbociclib combined with ET therapy did not translate short-term and PFS benefits into long-term OS prolongation despite PFS, ORR, and CBR benefits. For patients with HR+/HER2− EBC, abemaciclib improved IDFS in EBC compared with palbociclib but not in DRFS, and the results of the subgroup analysis suggested a significant IDFS benefit in combination therapy for early-stage high-risk patients with N2/N3 nodal stage. Due to the limited number of studies in RCTs and the immaturity of follow-up data, the evidence supporting combination therapy for EBC was not sufficient and the overall quality of evidence was low.

### Main findings

IDFS, which is the primary efficacy endpoint for EBC, is defined as the rate of invasive cancer recurrence after breast cancer treatment. The 2 MAs both pooled the IDFS of CDK4/6 inhibitors in combination with ET for EBC in the MonarchE (Johnston et al. 2020), PALLAS (Mayer et al. 2021), and PENELOPE-B trials (Loibl et al. 2021). The 2 MAs found that the IDFS benefits of the three clinical trials were more divergent and the MAs were somewhat controversial in terms of their clinical statistical benefits, subgroup analysis of lymph nodal stage and heterogeneity, so the results should be interpreted with caution. This may be related to the following reasons, but they are speculative. First, the underlying characteristics of the enrolled patients differed, such as the definition of high-risk patients and the proportion of the enrolled population. Second, the distinctions between CDK4/6 inhibitor medications, such as the MonarchE trial for abemaciclib and the PALLAS and PENELOPE-B trials for Palbociclib. In addition, differences in the pharmacological profile of different CDK4/6 inhibitors, different dosing regimens for continuous versus intermittent dosing, and differences in the duration of follow-up may lead to differences in the benefit of IDFS (Marra and Curigliano 2019). Notably, the IDFS benefit was primarily driven by data from the early MonarchE trial exploring abemaciclib in combination with ET for EBC (Agostinetto et al. 2021; Gao et al. 2021). In contrast, no IDFS benefit was observed in either the PALLAS or PENELOPE-B trials of palbociclib, which may be related to the toxicity and high treatment interruption rate of palbociclib. It is stressed that the publication of early MonarchE trial data in 2020 should be interpreted with caution. The median follow-up of the MonarchE trial in the early data was 15 months, and while the positive results showed that abemaciclib was effective in lowering the risk of invasive disease at 2 years—the primary outcome in this high-risk population, it was still unclear whether this benefit can be sustained in late recurrence and overall survival in patients with EBC, which might also add to the heterogeneity between MA data (Johnston et al. 2020). The intermediate follow-up data from the 2023 MonarchE experiment, however, were a positive finding. The absolute difference between abemaciclib and ET increased from 2.5% at 2 years of early follow-up to 5.9% at 4 years of follow-up, indicating a continuing deepening of the IDFS benefit after the termination of the treatment (Johnston et al. 2023). In addition, the analysis at mid-term OS found that the data in the abemaciclib group remain promising and were expected to further influence the difference in OS with additional follow-up. Therefore, to further clarify the role and evidence of CDK4/6 inhibitors in the adjuvant treatment of HR+/HER2− EBC, updated clinical trial data and the ongoing NATALEE trial still need to be explored (Johnston et al. 2023; Gnant et al. 2022; Rugo et al. 2022).

For HR+/HER2− ABC, there was broad agreement in the MAs findings that CDK4/6 inhibitors were associated with significant improvements in PFS, OS, ORR, and CBR, affirming the short- and long-term clinical efficacy of combination therapy for ABC. Notably, Munzone et al. (2021) looked at PFS2 and TTC outcomes and found that combination therapy improved PFS2 and TTC, which was consistent with the results of a MA published during the review of this study (Dai et al. 2022), suggesting that combination therapy may delay the onset of endocrine resistance and delay the duration of chemotherapy and chemotherapy-related toxicity, which may translate into a significant OS benefit and maintain a better quality of life for patients over a longer period of time.

Stratified exploration of patients according to their clinical and pathological characteristics classification, prior treatment history, and other factors contributes to clinical decision optimization for CDK4/6 inhibitors. Subgroup analysis revealed that combination therapy significantly improved PFS regardless of histopathological classification, endocrine resistance status, hormone receptor status, site and the number of tumor metastases, menopausal status, prior chemotherapy treatment, ET regimen, advanced disease treatment line, CDK4/6 inhibitor type, disease-free interval (DFI), and length of the treatment-free interval (TFI). Significant OS advantage was reported in the majority of subgroups in a stratified assessment of OS benefit, including hormone receptor status, menopausal status, the location and number of tumor metastases, age, race, line of disease therapy, and endocrine therapeutic agents. Notably, compared to ribociclib and abemaciclib, palbociclib in conjunction with ET did not significantly improve OS in subgroup analyses of CDK4/6 inhibitor types (Tian et al. 2021; Lin et al. 2020; Zheng et al. 2020; Piezzo et al. 2020). Although the three CDK4/6 inhibitors shared intrinsically similar patterns of activity as multikinase inhibitors, biological analysis contended that their unique chemical structures and disparate modes of action might account for the differences in clinical activity and survival of the various CDK4/6 inhibitors (Roberto et al. 2021; Chen et al. 2016). According to the aforementioned findings, even if the three CDK4/6 inhibitors all have a similar short-term PFS benefit, ribociclib and abemaciclib in conjunction with endocrine treatment are frequently more advantageous alternatives if the PFS benefit is to be converted into a long-term OS extension. It will need more prospective research to further support these findings over time.

### Evidence assessment

This comprehensive study used the AMSTAR-2 scale to assess the methodological quality of the included studies and identified several potential deficiencies: (1) the lack of a list of rejected literature and an explanation for its absence in all investigations reduced the reproducibility and transparency of the studies’ findings (Shen et al. 2021). (2) Basic aspects of the included studies, such as the study population, design, intervention/control measures (dose), follow-up activities, analysis procedures, and outcome indicators, were not well explained, and inadequate raw data could have exacerbated heterogeneity. (3) Since the funding source was not disclosed, it was difficult to tell whether the study’s planning, execution, and reporting were influenced by financial considerations. This made it difficult to determine whether the clinical trial results' objectivity, fairness, and authenticity were affected, which resulted in publication bias.

Particularly, this analysis employed the recently released PRISMA 2020 standards (Page et al. 2021a, b) for report quality assessment as opposed to the PRISMA 2009 recommendations (Hutton et al. 2015), which were used for the majority of MAs and SRs. Refining data items, data synthesis methods, study selection, data synthesis results, discussions, registries and protocols, the PRISMA 2020 guidelines reflect advances in methods for identifying, selecting, evaluating and synthesizing studies (Page et al. 2021a, b). The evaluation of the PRISMA 2020 statement for the included studies revealed that the incomplete search strategy, lack of methods of heterogeneity and sensitivity analysis in the methods and results, failure to explain the rationale for data exclusion in the study selection, discussion of any limitations of the review process not described, and failure to provide registration and protocols were the main reporting deficiencies and need to be further improved in the follow-up studies.

The GRADE approach was used to evaluate the included studies’ evidence quality, and it was discovered that the risk of bias, publication bias, and consistency issues were the main causes for the downgrading of the risk of bias. On a deeper level, implementation bias, measurement bias, blinding of participants and trial staff, and blinding of outcome assessment were the primary causes of the risk of the bias index being downgraded. In fact, this blinded flaw was mainly driven by the results of the PALOMA-1 trial (Finn et al. 2015), which also suggest the need for a standardized RCT. Furthermore, The MAs and SRs included in this study were performed on global multicenter clinical RCTs, which led to the creation of publication bias and reduced quality of evidence due to the limitations of the number of RCTs themselves.

### Limitations and strengths

There are some limitations to this review. In the first place, because this study was an analysis of pooled data, it could not be based on a data-level analysis of individual patients, which meant that this did not take into account separate confounding factors. Then, similar to many published MAs, an overemphasis on the positive nature of the findings may lead to the presence of publication bias, while the literature retrieved for this study was all in English, which might lead to selective bias.

In comparison, our study has some advantages. To the finest of our knowledge, this is the first umbrella review to comprehensively and critically summarize the evidence for CDK4/6 inhibitors in combination with ET for breast cancer. The comprehensive nature of this review is unique given the breadth of the assessment of clinical efficacy in EBC and ABC. First, it was using an umbrella review of studies that extensively summarized and summarized the clinical efficacy of CDK4/6 inhibitor combined with ET, which can improve the accuracy of the data among the many MAs and SRs of the results. In addition, the use of AMSTAR and GRADE assessment tools for uniform methodological and evidence-quality assessment of published MAs and SRs provided high-quality evidence for clinical decision-making and practice. The additional benefit of this study is that we developed strict inclusion and exclusion criteria based on the PICOS principles, and we also included only MAs and SRs using RCT study types, excluding literature using single-arm studies, non-randomized prospective, retrospective, and observational studies as study types, which reduced the interference of subjective factors and made the study results more replicable.

## Conclusion and prospect

According to this comprehensive review, a significant number of indicators of middle to high evidence were found to support that CDK4/6 inhibitor combined with ET significantly improved PFS, OS, ORR, and CBR in patients with HR+/HER2− ABC, demonstrating the validity of the findings, but palbociclib in combination with ET did not achieve long-term despite the ORR and PFS benefits obtained OS benefit. CDK4/6 inhibitors compared to palbociclib, abemaciclib improved IDFS in early high-risk breast cancer, but the quality of the evidence was low due to immature follow-up data in EBC.

In subsequent studies, the duration of continuous dosing of CDK4/6 inhibitors in EBC, how to administer cross-line therapy, timing of neoadjuvant chemotherapy, reduction of high discontinuation rates due to adverse events, and selection of cross-line regimens for ABC, and identification of biomarkers for CDK4/6 inhibitors are important subsequent developments in this field.

## Supplementary Information

Below is the link to the electronic supplementary material.

Supplementary file1 (DOCX 90 KB)

## Data Availability

The datasets used and/or analyzed during the current study are available from the corresponding author on reasonable request.
